# Huge uterine abscess after myomectomy: A case report

**DOI:** 10.1097/MD.0000000000037009

**Published:** 2024-01-26

**Authors:** Biwei Wen, Xiaomin Yu, Yue Yin, Runan Zhao, Yuhuan Liu

**Affiliations:** a Department of Obstetrics and Gynecology, The First Affiliated Hospital of Naval Medical University, Shanghai, China; b Department of Pathology, The First Affiliated Hospital of Naval Medical University, Shanghai, China.

**Keywords:** hysterectomy, MRI, PET/CT, uterine abscess

## Abstract

**Rationale:**

Uterine abscess is a rare gynecologic entity and only a few cases have been reported so far. This study aimed to describe our clinical experience in this case. Initially, hematoma was diagnosed without detail previous medical record. Finally, laparotomy was performed due to refractory fever and highly possible diagnosis of uterine abscess. We successfully performed a hysterectomy and the patient had an uneventful recovery.

**Patient concerns:**

A 44-year-old nulliparous woman underwent myomectomy in the local hospital, 45 days ago. She complained of irregular fever (up to 40 °C) without abdominal pain since the surgery.

**Diagnoses:**

Due to lack of her detail medical record, equivocal images and her strong intention to preserve uterus, she was misdiagnosed with hematoma and treated with antibiotic treatment. Finally, intraoperative findings revealed that the huge myometrial abscess contained a mass of pus.

**Interventions:**

Laparotomy was performed due to refractory high-grade fever and highly possible diagnosis of uterine abscess. Total hysterectomy was performed to avoid the possibility of life-threatening sepsis.

**Outcomes:**

The postoperative course was uneventful and the patient was discharged 10 days after surgery.

**Lessons:**

Complete imaging examinations are recommended prior myomectomy to facilitate the differential diagnosis of postoperative complications. In addition, several measures, such as maintaining aseptic conditions during surgery and postoperative drainage, play a critical role in preventing nosocomial infections. Rare uterine abscess is often mistaken for hematoma with fever. If the patient develops high fever after myomectomy, accompanied by a mass in the myometrium, the possibility of infection or even abscess formation should not be excluded. For women who need to preserve their fertility, the early diagnosis and timely administration of appropriate medication is crucial for preventing uterine loss.

## 1. Introduction

Uterine abscess is a rare form of severe inflammation. Myomectomy is a very common gynecological surgery. Uterine abscess, which occurs only in the myometrium immediately after myomectomy, is even rarer. In our case, a huge uterine abscess after myomectomy was reported. Furthermore, the process of diagnosis and treatment was analyzed and measures to prevent such catastrophic events were suggested.

## 2. Case report

**Figure 1.**
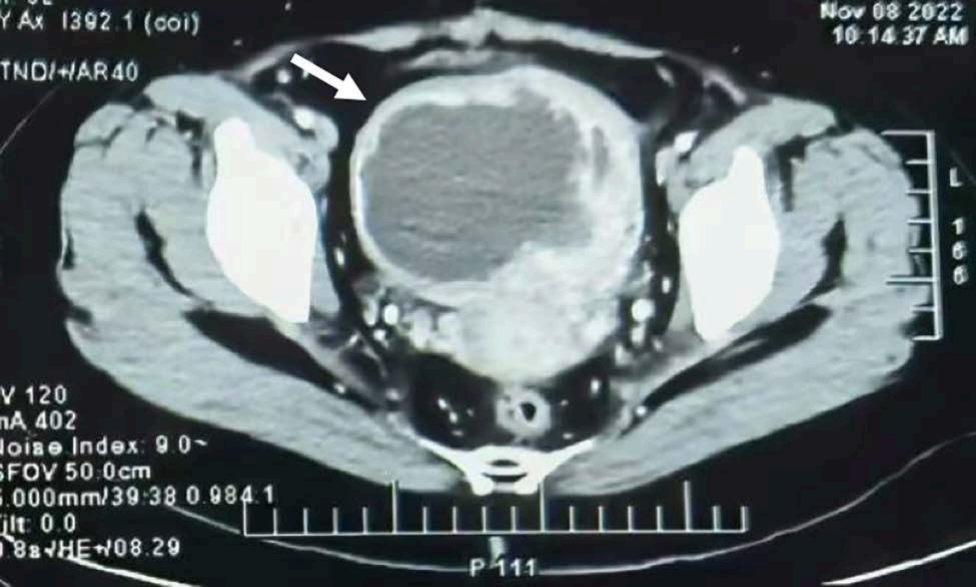
Enhanced CT scans showed an abnormal mass in the uterus. CT = computed tomography.

A 44-year-old nulliparous woman underwent myomectomy in the local hospital 45 days ago. According to her medical history, preoperative ultrasound showed an 8-cm myoma in the posterior wall of the uterus. However, the surgery record described that the uterus was approximately 24 weeks’ gestation and the 8-cm in diameter myoma was resected from the anterior wall of the uterus. No further details about the operation were provided. The patient complained of irregular fever (up to 40 °C) without abdominal pain since the surgery. She had no history of endometriosis, pelvic inflammatory disease or other surgery, and her family history was unremarkable. Enhanced computed tomography (CT) scans showed an abnormal mass in the uterus (Fig. 1), while transvaginal ultrasonography revealed a hematoma in the anterior wall of the uterus.

At the time of admission to the clinic, the patients’ vital signs were as follows: blood pressure, 105/66 mm Hg; heart rate, 90 bpm; and temperature, 37.4 °C. The size of her uterus was approximately 12 weeks’ gestation, which was bulky and mobile. No uterine, cervical or bilateral adnexal tenderness was recorded. Vaginal discharge was also normal. In addition, C-reactive protein levels were 17 mg/L, white blood cell (WBC) count was 12.6*10^9^/L, while procalcitonin levels were normal. Since suturing the myoma bed is considered as one of the most challenging aspects of myomectomy, the patient was diagnosed with hematoma with infection and she was, therefore, treated with oral antibiotics (levofloxacin).

**Figure 2.**
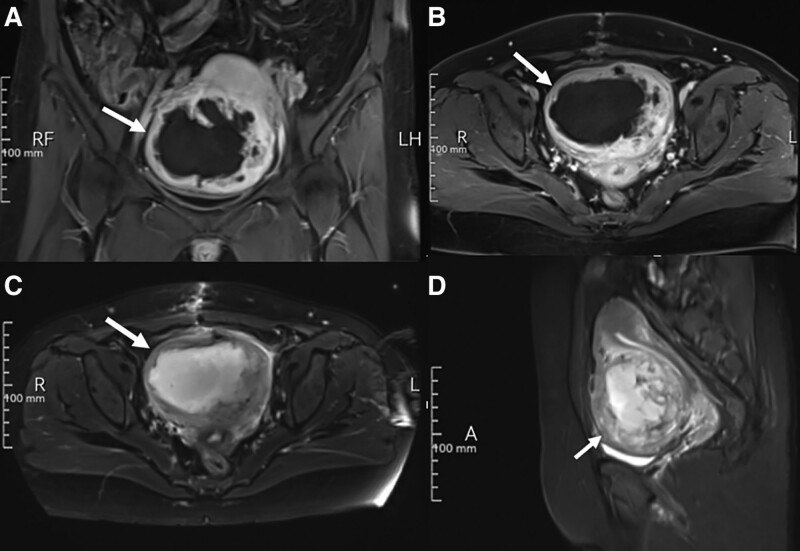
MRI scan revealed an 8-cm in diameter mass with thick wall in the anterior myometrium. The mass was solid-cystic (arrow), with mixed low intensity on T1-weighted (fat suppressed) images (A and B) and mixed high intensity on T2-weighted images (C and D). MRI scan suggested leiomyoma degeneration, along with necrosis and infection of the surrounding tissue. MRI = magnetic resonance imaging.

Magnetic resonance imaging (MRI) scan revealed an 8-cm in diameter mass with thick wall in the anterior myometrium (Fig. 2). The mass was solid-cystic, with mixed low intensity on T1-weighted images and mixed high intensity on T2-weighted images. MRI scan suggested leiomyoma degeneration, along with necrosis and infection of the surrounding tissue. Several routine laboratory tests were also performed after hospitalization, including discharge culture, assessment of serum tumor markers and thoracic CT scan. No positive results were obtained. Autoimmune diseases were also excluded due to negative antibodies.

**Figure 3.**
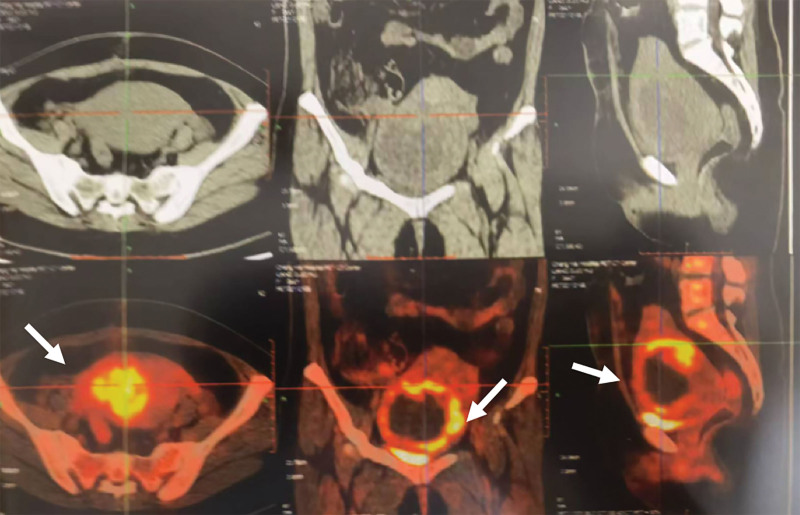
PET-CT scan showed an irregular mass in the anterior wall of the uterine, along with surrounding intensive ^18^F-fluorodeoxyglucose uptake (SUVmax of 5.8), without enlarged lymph nodes.

However, 5 days after admission, the woman developed fever (up to 39.4 °C) again, still without any abdominal and pelvic tenderness or pain. C-reactive protein and procalcitonin levels were still slightly elevated, with values of 20 mg/L and 0.123 ng/mL, respectively. WBC count was 15.2*10^9^/L and blood culture was still negative. In addition, positron emission tomography-CT scan also showed ^18^F-fluorodeoxyglucose uptake in the anterior wall of the uterine, without enlarged lymph nodes (Fig. 3). The possibility of hematopoietic system disease, such as lymphoma, was ruled out.

**Figure 4.**
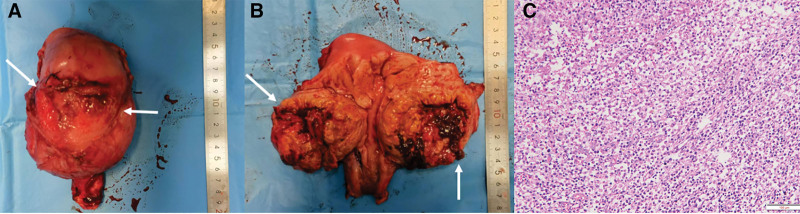
Uterus and anterior wall were markedly enlarged (arrow) (A). Macroscopically, an abscess (arrow), 8 cm in diameter, was identified in the anterior myometrium (B). Microscopically, a marked infiltration of foamy histiocytes, along with abundant lymphocytes and plasma cells was observed in the anterior uterine myometrium (C).

As the symptoms of the patient worsened, the diagnosis of a uterine abscess was considered. Therefore, the patient was treated with intravenous broad-spectrum antibiotics (vancomycin and imipenem) for one week. However, the high fever was persistent and the size of the uterus increased (16 weeks’ gestation). Therefore, conservative treatment was considered difficult and ineffective. After multidisciplinary discussions, a laparotomy was performed. Intraoperative findings revealed dense adhesions around the anterior wall of the uterus and bladder (Fig. 4A). Additionally, the uterus and anterior wall were markedly enlarged, tightly filling the pelvic cavity. Following perforation of the anterior wall of the uterus, 200 mL of pus was poured. No other lesions, such as pelvic inflammation, appendicitis or enlargement of lymphatic nodes were observed. After full discussion with her husband regarding the surgical plan, total hysterectomy was performed to avoid the possibility of life-threatening sepsis. Bilateral adnexa were reserved to preserve ovarian function. All cultures performed were sterile.

Macroscopically, an abscess, 8 cm in diameter, was identified in the anterior myometrium (Fig. 4B). The cavity of the abscess was purulent with necrosis and separated from the endometrium and perimetrium. No exudate was found on other surfaces. The endometrium was thin and the uterine cavity was empty. Microscopically, a marked infiltration of foamy histiocytes, along with abundant lymphocytes and plasma cells was observed in the anterior uterine myometrium. Furthermore, neutrophils were found at the anterior wall surrounding the abscess cavity (Fig. 4C).

The postoperative course was uneventful and the patient was discharged 10 days after surgery.

## 3. Discussion

Uterine abscess, which occurs only in the myometrium after myomectomy, is rare. It can be caused by invasive operation of the uterus or retrograde spread, like surgical wound infections penetrating the myometrium, associated with egg retrieval, cesarean section or pyometra.^[1–3]^ In the current case report, an abscess was identified only in the myometrium, without pelvic inflammation. Histopathological examination revealed only a pus-filled cavity intraoperatively. However, the discharge culture was negative, indicating that the abscess could not have spread from other tissues or organs, but originated from the mesometritis. Microscopically, an accumulation of WBCs, accompanied by foam cell infiltration, was observed in the area of adhesion between the uterus and bladder. The above WBC accumulation was not found in any other region of the uterine perimetrium. Although the microbe responsible for the infection was not identified, the aforementioned findings indicated that a previous myomectomy-induced injury and infection could had predisposed the patient to the development of an abscess.

In our case report, this woman did not undergo full imaging evaluation prior to myomectomy, such as pelvic MRI or CT scan. And she only presented with fever, without abdominal pain, which increase the difficulty of the diagnosis.

PET-CT may be helpful for detecting abscess, it usually shows the increased FDG uptake. The mean SUVmax of 9.4 in the tubo-ovarian abscess,^[4]^ 12.7 in urachal abscess,^[5]^ and 17.4 in cervical cancer, respectively.^[6]^ The sensitivity of diffusion-weighted imaging -combined MRI for diagnosing abscess has also been demonstrated in a few studies.^[7]^

Although, surgical drainage is considered a critical part of the treatment.^[8,9]^ Considering the high risk of bleeding and spread of infection through percutaneous drainage, conservative treatment was initially chosen. The patient was treated with broad-spectrum antibiotics for 1 week. However, surgery was performed due to severe high fever.

## 4. Conclusions

Successful treatment of uterine abscess can be achieved through initial diagnosis, empirical broad-spectrum antibiotic treatment, and even surgery. Preoperative imaging and maintenance of complete medical records are crucial for aiding diagnosis. Additionally, the role of multidisciplinary cooperation was also highlighted.

## Acknowledgments

The authors express their gratitude to the patient who made this work possible, as well as the professionals and researchers that participated in this study. A patient’s informed consent was acquired.

## Author contributions

**Conceptualization:** Biwei Wen.

**Supervision:** Yuhuan Liu.

**Validation:** Xiaomian Yu.

**Visualization:** Yue Yin, Runan Zhao.

**Writing – original draft:** Biwei Wen.

**Writing – review & editing:** Yuhuan Liu.

## References

[1] Poomalar GK Sasikala R Nivedita K. A case report of pyoperitoneum due to rupture of intramyometrial abscess in non-gravid uterus. J Obstet Gynaecol. 2020;40:584–5. 31339404 10.1080/01443615.2019.1614546

[2] Milman T Shivji A Edwards D. Myometrial abscess in the noninstrumented uterus. J Minim Invasive Gynecol. 2022;29:190–2. 34748967 10.1016/j.jmig.2021.10.015

[3] Liu Y. Actinomycosis-induced adnexal and uterine masses mimicking malignancy on FDG PET/CT. Am J Obstet Gynecol. 2019;220:281–28. 30096319 10.1016/j.ajog.2018.08.003

[4] Fan H Wang TT Ren G. Characterization of tubo-ovarian abscess mimicking adnexal masses: comparison between contrast-enhanced CT, ^18^F-FDG PET/CT and MRI. Taiwan J Obstet Gynecol. 2018;57:40–6. 29458901 10.1016/j.tjog.2017.12.007

[5] Flaus A Longo MG Dematons M. ^18^F-FDG PET/CT in urachal abscess. Clin Nucl Med. 2019;44:e349–50. 30829865 10.1097/RLU.0000000000002524

[6] Dhomps A Trecourt A Tordo J. Cervix abscess mimicking cervical cancer explored with 18F-FDG PET/CT and MRI. Clin Nucl Med. 2023;48:e237–8. 36728141 10.1097/RLU.0000000000004561

[7] Chun CW Jung JY Baik JS. Detection of soft-tissue abscess: comparison of diffusion-weighted imaging to contrast-enhanced MRI. J Magn Reson Imaging. 2018;47:60–8. 28432835 10.1002/jmri.25743

[8] Han SY Ryu KJ Ahn KH. Conservative treatment of uterine fistula with abdominal abscess after caesarean section. J Obstet Gynaecol. 2015;35:650–1. 25496617 10.3109/01443615.2014.987115

[9] Thanos L Tsagouli P Eukarpidis T. Computed tomography-guided drainage of a corpus cavernosum abscess: a minimally invasive successful treatment. Cardiovasc Intervent Radiol. 2011;34:217–9. 20596709 10.1007/s00270-010-9923-x

